# Learning motor actions via imagery—perceptual or motor learning?

**DOI:** 10.1007/s00426-022-01787-4

**Published:** 2023-01-21

**Authors:** Cornelia Frank, Sarah N. Kraeutner, Martina Rieger, Shaun G. Boe

**Affiliations:** 1 Department of Sports and Movement Science, School of Educational and Cultural Studies, Osnabrück University, Osnabrück, Germany; 2 Department of Psychology, University of British Columbia, Okanagan, Kelowna Canada; 3 Djavad Mowafaghian Centre for Brain Health, University of British Columbia, Vancouver, Canada; 4 Institute for Psychology, UMIT Tirol – Private University for Health Sciences and Health Technology, Hall in Tyrol, Austria; 5 Laboratory for Brain Recovery and Function, School of Physiotherapy, Department of Psychology and Neuroscience, School of Health and Human Performance, Dalhousie University, Nova Scotia, Canada

## Abstract

It is well accepted that repeatedly imagining oneself acting without any overt behavior can lead to learning. The prominent theory accounting for why imagery practice is effective, motor simulation theory, posits that imagined action and overt action are functionally equivalent, the exception being activation of the end effector. If, as motor simulation theory states, one can compile the goal, plan, motor program and outcome of an action during imagined action similar to overt action, then learning of novel skills via imagery should proceed in a manner equivalent to that of overt action. While the evidence on motor simulation theory is both plentiful and diverse, it does not explicitly account for differences in neural and behavioural findings between imagined and overt action. In this position paper, we briefly review theoretical accounts to date and present a perceptual–cognitive theory that accounts for often observed outcomes of imagery practice. We suggest that learning by way of imagery reflects perceptual-cognitive scaffolding, and that this ‘perceptual’ learning transfers into ‘motor’ learning (or not) depending on various factors. Based on this theory, we characterize consistently reported learning effects that occur with imagery practice, against the background of well-known physical practice effects and show that perceptual-cognitive scaffolding is well-suited to explain what is being learnt during imagery practice.

To imagine an action such as a golf putt, to ‘see’ the ball, to ‘feel’ the club, to ‘hear’ the club head-ball-impact, is one of many fascinating capacities of human beings. What is even more intriguing is that we can learn by way of imagined actions. Like *mental imagery* being a multisensory vicarious experience (Lacey & Lawson, 2013), *imagery in sport* is a (re-) creation of experience in the absence of the actual sensory stimulus (Annett, 1995, 1996; Farah, 1984; Morris et al., 2005). *Imagined action* (vs. *overt action*)^1^ relates to the imagery of one’s own action without any overt behavior, and with the imager being the agent of the action (Jeannerod, 1995; Munzert & Zentgraf, 2009). *Imagery practice* (i.e., *mental practice; imagery training; action imagery practice*) denotes the imagery of an action in a systematic and repetitive manner, and *physical practice* relates to the systematic and repetitive use of overt action. While syntheses of evidence to date have shown that we can learn from imagining motor actions without executing them, the mechanisms that lead to learning via imagined action are still being debated. The aim of the position paper is to delineate a perceptual-cognitive theory for imagery practice effects. The new account helps to better understand imagery practice effects by explaining how imagined action impacts performance and learning. From a broader perspective, it serves to shed additional light on the relation between cognition and movement, here between imagined action and the learning of a motor action.

## Learning via imagined action – similar to that of overt action?

Imagery practice can improve motor performance and promote motor learning (e.g., Corbin, 1967; for reviews and meta-analyses, see Driskell et al., 1994; Feltz & Landers, 1983; Feltz et al., 1988; Richardson, 1967; Simonsmeier et al., 2020; Toth et al., 2020). In this sense, imagery practice and physical practice are similar in that both can improve performance and induce learning﻿: Meta-analyses have shown small to medium effect sizes for imagery practice (Driskell et al., 1994: 35 studies/62 effect sizes/d = 0.527; Toth et al., 2020: 37 studies/99 effect sizes: d = 0.419/d = 0.264 [after publication bias correction]; Simonsmeier et al., 2020: 48 studies/ 304 effect sizes/d = 0.416). Thus, if one imagines a motor action in a systematic manner, imagery practice is likely to bring about changes in performance and in some instances learning. However, imagery practice effects are usually lower in magnitude compared to physical practice effects (Corbin, 1967; Frank et al., 2014; Ingram et al., 2019; Kraeutner et al., 2020b). In contrast, if performed in combination while controlling for the overall amount of practice, effects of combined imagery and physical practice on motor performance and motor learning are even greater than of physical practice alone (McBride & Rothstein, 1979; Simonsmeier et al., 2020).

Among the explanations why imagery practice may be effective (e.g., Jacobson, 1931; Sackett, 1934; Heuer, 1985, 1989; Schack, 2006), motor simulation theory and has received strong support (Jeannerod, 1994, 2001). Central to motor simulation theory is the principle of equivalence between imagined and overt action (Finke, 1979; Jeannerod, 1994, 1995; Johnson, 1982). Imagined action is suggested to be ‘functionally equivalent’ to overt action in that both states share similar processes and draw on the same action representation, with descending motor commands being inhibited in imagery (see Grospetre et al., 2016; Guillot et al., 2012; Kasess et al., 2008; Solomon et al., 2019; for a detailed account of mechanisms) and thus not leading to any observable action compared to overt action (Jeannerod, 1995, 2001). Within this context, imagery is predicted to elicit neural activity in motor-related areas of the brain shared with overt action and, therefore, allows for learning. Consistent with this theory, many studies have shown similar brain activity in imagined and overt action, suggesting a ‘structural equivalence’ between the two states (Burianova et al., 2013; Kraeutner et al., 2014); indeed, meta-analysis of neuroimaging experiments (Hetu et al., 2013) indicates that imagery activates frontal premotor and parietal regions of the brain in a similar way to overt action. In addition to the similarities predicted by motor simulation theory and given that imagery as a covert state does not involve an overt state of action, imagined action should differ from overt action in exactly the processes that accompany the overt stage of action and, therefore, are lacking during imagery (Munzert et al., 2009). Indeed, and in addition to neural similarities, differences in brain activation have been reported between imagined and overt action. For instance, Zabicki and colleagues (2017) found a similar structural geometry in brain activation for imagined and overt hand actions, but with the best model fit for low-to-moderate degree of similarity. In a recent meta-analysis, Hardwick and colleagues (2018) demonstrated that the volume activated during imagined action was less than half of that during overt action and that regions related to action preparation were consistently recruited during imagined action, but not during overt action. Along these lines, activation of the primary motor cortex, present for overt action, is not consistently observed during imagined action, and if present, is notably lower in magnitude (Hardwick et al., 2018; Hétu et al., 2013). These and other findings show that imagined action recruits partly similar, partly distinct brain areas (for reviews, see Hardwick et al., 2018; Ladda et al., 2021).

In sum, although learning via imagined action is similar to learning via overt action in the sense that it induces action-related changes, learning via imagined action is different. First, as detailed above, while overlap with overt action exists, imagined action recruits different brain regions (for details on perceptual components of movement/ fronto-parietal networks, see section ‘Evidence for perceptual-cognitive scaffolding’). Second, although completing the same amount of practice, imagery practice is in most cases less effective in behavioral terms than physical practice. Third, when combining imagery practice and physical practice, the magnitude of behavioural effects outperforms that of physical practice. Superadditive effects like these cannot be explained by theories that consider imagined action and overt action equivalent. Collectively, this evidence indicates differences in underlying processes, neural underpinnings and behavioural outcomes of imagined and overt action. This raises the question as to whether unique processes characterize the two states of action.

## Understanding learning via imagined action—Theories to explain imagery practice effects

Theories that aim to explain imagery practice effects come from a variety of disciplines such as cognitive psychology (MacKay, 1981; Sackett, 1934), psychophysiology (Jacobson, 1931), neuroscience (Jeannerod, 1994) and movement science (Frank, 2014; Heuer, 1985, 1989; Schack, 2006). To date, it is commonly agreed that imagery practice effects are specific, and not only of motivational or of modelling nature (Heuer, 1989; Mendoza & Wichman, 1978). The theories and models that refer to the specific effect of imagery on the motor action system have focused on different aspects such as the nature of the task (cognitive vs. motor; Ryan & Simons, 1983), the role of executive functions (Glover et al., 2020), the location of effects (central vs. peripheral; Jacobson, 1931), the level of effect (effector-dependence vs. -independence: Dahm et al., 2022; Ingram et al., 2016; Kraeutner et al., 2017; Mizuguchi et al., 2014),or a shared representational format (﻿Annett, 1996; Heuer, 1989; Jeannerod, 1995; Schack, 2004).

Aimed at formalizing the exact relation between cognitive processes and movement, here imagery and the learning of a motor action, approaches vary in the nature of the specific relation hypothesized between imagery and performance and imagery practice and learning. For instance, Heuer (1985, 1989) elaborated on a correlational relationship between imagery and performance/learning. According to his multiple representation perspective, humans can learn any representational format. Whether imagery leads to motor learning crucially depends on whether the transformation between different representations is possible, that is, whether transformation rules between visuospatial and kinesthetic representations have been learnt yet (i.e., exist). More recently, motor simulation theory (Jeannerod, 1994, 2001) suggests that learning by way of imagined action is possible to the extent to which an overlap in neural representations between imagined and overt action exists. Testing the idea of a higher order (less direct) relation against a first-order (direct) isomorphic relation between images and the percepts they represent (for details, see Shepard & Cooper, 1982), Coelho and colleagues (2012) showed that typical scheduling effects in physical practice do not hold for imagery practice. While variable (overt) practice of a golf putt was superior to constant (overt) practice, this did not hold for imagery practice, indicating subtle differences between imagined and overt action (and between images and related percepts), resulting in differences in learning by way of imagined vs. overt action.

Despite the longstanding history of imagery practice, the growing evidence in the field, and the variety of theories seeking to explain imagery practice effects (for an overview, see e.g., Frank, 2014; Heuer, 1985, 1989; Kraeutner, 2019; Morris et al., 2005; Murphy, 1990; Murphy et al., 2008; Schack, 2006), the debate about how imagery practice works persist until today. How does imagery impact performance, and what is the exact relation between cognitive processes and movement, here between imagery practice and the learning of motor action?

## Learning via imagined action through perceptual–cognitive scaffolding

In this position paper, we argue for a perceptual–cognitive scaffolding approach and why it can explain learning-related evidence to date. We suggest that learning by way of imagery reflects perceptual-cognitive scaffolding, and that this ‘perceptual’ learning transfers into ‘motor’ learning – or not – depending on various factors.

According to perceptual-cognitive approaches, actions are primarily guided by cognitively represented perceptual effects (e.g., e.g., Hoffmann et al., 2004; Hommel et al., 2001; Jeannerod, 2001; Knuf et al., 2001; Kunde, 2001; Schack, 2004). Ideomotor theory posits that a link between the action and its effects is established during learning (ideomotor theory: James, 1890; learning: Hommel & Elsner, 2009; Wulf & Prinz, 2001; Ziessler & Nattkemper, 2002; Ziessler et al., 2004). Along these lines, motor actions can be considered as being stored and represented as perceptual-cognitive networks that guide action control (Schack, 2004, 2020).

Based on the assumption that both imagined and overt action draw on the same representation and involve similar albeit possibly distinct processes (Jeannerod, 1995; Munzert et al., 2009; Schack & Frank, 2019), imagery practice has been suggested to be effective because it draws on a perceptual-cognitive representation and refines the representational networks of action organization (Frank, 2014; Schack, 2006; Schack & Frank, 2019). According to the perceptual-cognitive hypothesis (Schack, 2006), imagery practice is effective because it links cognitive representations to perceptual ones in a hierarchy of mental and sensorimotor levels of action organization (cf. cognitive action architecture approach/CAA-A; for an overview, see Schack, 2004, 2020). While the perceptual-cognitive hypothesis emphasizes the central role of representations, it remains unclear whether imagery and execution are similar or different in driving learning (for proposed differential effect within action hierarchy; see, Frank, 2014). To address this gap, we suggest perceptual-cognitive scaffolding as core process that drives the learning by way of imagined action.

During imagery practice, a perceptual-cognitive action representation is activated, manipulated and stabilized (Farah, 1984; Schack & Frank, 2019). Accordingly, and assuming a close linkage between anticipated action effects and imagery (Bach et al. in this issue), learning by way of imagined action is possible because repeated anticipation of action effects during goal-directed imagined action leads to gathering, structuring and fostering of (quasi^2^-)action effects. This perceptual-cognitive scaffolding, in turn, helps to guide future action.^3^ Perceptual-cognitive scaffolding thus emphasizes the process of actively building a cognitive scaffold from action-related quasi-perceptual effects. During this process, the imager both recreates effects from his/ her experience and creates/estimates effects based on his/her experience. Based on perceptual-cognitive representations in long-term memory, quasi-perceptual effects are being manipulated in working memory whilst imagining in a goal-directed manner, and this manipulation leads to changes in perceptual-cognitive representations in long-term memory again. Given that this assembling of effects is not based on overt action and real experience (as it is during execution), scaffolding denotes the tentative nature of setting up and shaping the learner’s representation through this action-related and goal-directed process. This process may or may not involve (quasi-) feedback from imagined action (for a discussion, see Rieger et al. in this issue). The resulting scaffold may not be the final and most appropriate one for a given action, but helps the learner as an estimate, a frame or a model, for future (overt) action control.

A perceptual-cognitive scaffold is thus a platform or foundation that contains information of (quasi-)action effects/anticipated (quasi-)sensory consequences for which motor commands can then be readily produced. The process of scaffolding thus represents the gathering, structuring and fostering of cognitively represented perceptual effects of the action which results in a refined representation that guides one’s actions. Importantly, and in contrast to the programming hypothesis (Heuer, 1985, 1989), learning by way of imagery may not be caused by specification of motor commands in the first place, but rather by specification of perceptual effects during imagery. This repeated imagery/ anticipation of action effects fosters action-related effects and leads to perceptual-cognitive scaffolding, which guides future action and can lead to improved behavior in the sense of more successful motor planning and execution.

Whether or not this perceptual-cognitive scaffolding directly transfers to improved overt action should depend on whether the link between the action and its effects exists and how strong the link is. For instance, in the case of unskilled action, perceptual-cognitive scaffolding should not transfer into improved overt action, due to a missing link between an action and its imagined effects. From an effect-based view of action control, only if representations stored in long-term memory are associated with the relevant motor activities that reliably produce the intended effects would learning by way of imagery become evident as improvements in overt behavior. This may of course as well be the case, if one draws on experience of similar tasks, and thus representations of related and transferable actions (e.g., imagining to type in an unfamiliar style may draw on the experience one has with typing in the usual typing style; Rieger, 2012). Such transfer effects can explain why novices still can show some minor improvement in overt action, as some aspects of the action to be learnt can be transferred and built from related experience.

From a perceptual-cognitive scaffolding point of view, the following should be observed: (1) Imagery practice effects should depend on the type of task, with cognitive tasks profiting more from perceptual-cognitive scaffolding, as little to no transfer to motor levels of action control is necessary; (2) Imagery practice effects should depend on skill level; it should be more difficult to learn truly motor tasks from perceptual-cognitive scaffolding in cases when the imager has no overt, physical experience (and thus no representation of kinesthetic aspects, the feel of the movement, bodily effects of the action). Imagery should not affect performance in the case of true novices. It should though be effective for imagers with experience, as experts, for instance, know the relation between an action and its effects, and thus should be able to imagine appropriate effects that cause functional changes in action representation; (3) Transfer effects from tasks that share action effects and thus perceptual-cognitive scaffolds should be possible; similarly, performance after imagery practice should decline if perceptual components are changed in a perceptual transfer task; (4) Imagery instructions focusing on the most relevant perceptual aspects of the task to be learnt should boost practice effects; (5) If imagery practice and physical practice are different, then combinations should lead to superadditive effects; similarly, scheduling effects observed in physical practice must not necessarily hold for imagery practice (for more details, see Section ‘When is imagery more or less effective?’).

## Evidence for perceptual-cognitive scaffolding during imagery practice

Research conducted in the realm of the cognitive action architecture approach (CAA-A; for a review, see Schack, 2020; Schack et al., 2014) supports the idea of perceptual-cognitive scaffolding and related changes in action representation. Based on the finding that experts hold structured representations with functional groupings of action-related perceptual-cognitive sub-units (Bläsing et al., 2009; Schack & Mechsner, 2006), novices' unstructured representations functionally change during motor learning (Frank et al., 2013). Novices who repeatedly executed the golf putt over the course of 3 days held structured action representations with functional groupings after practice. Specifically, their representations revealed a structure that reflected key parts of movement phases pertaining to the functional and biomechanical demands of the task (e.g., preparation, clubhead-ball impact, and an attenuation phase; Frank, 2016; Frank et al., 2013). Likewise, imagery practice has been shown to change perceptual-cognitive representation structures in long-term memory (for an overview, see Frank & Schack, 2017). When novices practiced by way of imagery, their perceptual-cognitive representation structures were more similar to a functional structure compared to novices who did not incorporate imagery (Frank et al., 2014, 2016), indicating that a perceptual-cognitive scaffold has developed. However, imagery practice and related representational changes do not necessarily transfer into changes in motor behavior (until overtly executing a task) due to a missing link between an action and its effects in novices.

Further evidence comes from research on implicit sequence learning. Boe and colleagues have shown that learning via imagery relies more on perceptual than on motor aspects of the action (e.g., Ingram et al., 2016, Ingram, 2019; Kraeutner et al., 2017). Testing the hypothesis that learning via imagined action may be based on perceptual rather than motor learning, Ingram and colleagues (2016) compared mental to physical practice and the transfer of learning when altering perceptual or motor aspects of the task. Alongside of their hypothesis, they found that altering the sensory cue (i.e., visual vs. auditory) had a greater disruptive effect on reaction times after imagery practice compared to physical practice. This indicates that performance improvements via imagined action may rely more on perceptual learning. Using the same implicit sequence learning paradigm, Kraeutner, MacKenzie and colleagues (2016) reported that while the magnitude of the difference between random and repeated sequence elements was similar following practice via imagined and overt action, a general effect of practice via overt action was observed such that reaction times to both random and repeated sequence elements was faster than that observed following practice via imagined action. That a general effect of practice was found for overt action but not imagery indicates that imagery involves more perceptual learning, as mapping of a perceptual stimulus to a motor response (i.e., stimulus–response mapping) via imagined action was as effective as in overt action, but no effect on the motor component of performance was observed for imagined action, whereas such an effect was observed for overt action. Evidence for perceptual learning in action imagery was also found with an explicit sequence learning task using intermanual transfer tests (Dahm et al., 2022). In this study, participants learned to sequentially move with one finger to ten targets, which were visible the whole time in four practice sessions. In both imagery practice and physical practice, movement times were significantly shorter in the practice sequence than in the other sequence in the transfer hand, which indicates effector-independent visual-spatial learning. Further, in physical practice, but not in action imagery practice, movement times were significantly shorter in the practice hand than in the transfer hand, indicating effector-dependent learning in physical practice only.

Neuroscientific evidence to date indicates that changes in action representation primarily relate to perceptual-cognitive rather than motor aspects (Avanzino et al., 2015; Jackson et al., 2003; Olsson et al., 2008; Pascual-Leone et al., 1995; Ruffino et al., 2019; Zhang et al., 2014). For instance, Kraeutner and colleagues (2022), using resting state functional magnetic resonance imaging to examine changes in brain activity occurring during learning, showed that imagery practice drives greater functional changes in a frontoparietal network relative to physical practice. Using a finger tapping task, Olsson et al. (2008) found increased activation in brain regions specific to the type of practice used, as revealed by changes in motor regions after physical practice and changes in visual regions after imagery practice. Interestingly, combined mental and physical practice led to improved transfer effects on the performance level that were associated with activation in the cerebellum. They concluded that learning via imagined action may result in the generation of abstract representations and that these cognitive changes may only transform into motor programs through cerebellum activation when combined with physical practice.

Of relevance to the notion of perceptual-cognitive rather than motoric aspects of action resulting from imagery is the importance of fronto-parietal networks, critical to more transformative and visuospatial processes that support motor performance, to imagery performance and learning. Stemming from pioneering work from Sirigu and colleagues (1996) who showed impairment in the ability to generate a representation of hand following parietal cortex damage, numerous studies have shown fronto-parietal (Oostra et al., 2016) or parietal (McInnes et al., 2015) lesions to impair imagery performance. Moreover, inhibition of the inferior parietal lobe (involved in perceptual integration and visuomotor processes), but not primary motor cortex (attributed to core motor processes including outputting the motor command to the effectors), by non-invasive brain stimulation (transcranial magnetic stimulation, TMS) impairs imagery performance and learning of novel sequences by imagined action, but not physical performance (Kraeutner et al., 2016a, 2016b, 2017, 2019). Further, the role of the IPL in imagery has been demonstrated in mental rotation tasks. Related work (Hamada et al., 2018; Kosslyn et al., 1998) showed greater activation in posterior parietal regions when performing mental rotation of hands (for which imagined action is required to solve the problems related to the orientation of hands presented on a screen) vs. mental rotation of objects (e.g., cubes and cars, for which imagined action is not required). While the greater activation and reliance on fronto-parietal regions in imagined action relative to overt action is attributable in part to the generation of an image, the evidence presented above supports the notion that imagery processes are multidimensional (i.e., beyond just the generation of an image, but also its maintenance and manipulation; Cumming & Eaves, 2018; Kraeutner et. al., 2020c; Ptak et al., 2017). Further, that fronto-parietal networks are modulated by imagery-based practice (see Kraeutner et. al., 2022) suggests imagery is affecting processing that occurs at the perceptual-cognitive level.

In sum, increasing evidence indicates that changes take place on perceptual-cognitive levels of motor action, both in learning of complex action as well as in sequence learning. While the principle of functional equivalence and the motor simulation theory cannot fully explain differences in learning as a result of imagery practice or physical practice, these differences can be explained through a perceptual-cognitive scaffolding lens.

## When is imagery practice more or less effective? Revisiting influential factors from a perceptual-cognitive scaffolding perspective

Empirical findings from imagery research to date have repeatedly shown that imagery practice effects depend on various factors such as task, instruction, skill level, and scheduling. In the following, we briefly review factors that influence imagery practice effects and interpret the diversity of findings about learning by way of imagined action from a perceptual-cognitive scaffolding perspective.

**(1) On the influence of the task to be learnt**: ***Imagery practice has proven to be more effective for ‘cognitive’ tasks; for ‘motor’ tasks, imagery practice leads to functional changes on perceptual-cognitive levels of action organization.***

The nature of the task used for imagery practice and thus the task to be learnt varies tremendously across studies, being categorized into cognitive tasks (e.g., Sackett, 1934) vs. motor tasks (Jacobson, 1931; for cognitive-motor hypothesis, see Ryan & Simons, 1983), coordination tasks (e.g., White & Hardy, 1995) vs. strength tasks (e.g., Reiser, 2005), and single tasks (e.g., Mendoza & Wichman, 1978) vs. sequential tasks (Wohldmann et al., 2007). Imagery is more effective for cognitive tasks compared to motor tasks (e.g., Driskell et al., 1994; Minas, 1978, 1980; Ryan & Simons, 1981, 1983). For instance, investigating the cognitive-motor hypothesis, Ryan and Simons (1981, 1983) compared mental and physical practice effects in motor tasks high in motor components and tasks high in cognitive components (e.g., balance vs. maze task; maze task with high and low motor component) and found that practice effects did not differ in tasks with high cognitive demands, while physical practice was superior to mental practice in tasks with high motor demands. Along these lines, imagery practice has proven to be effective in sequence learning (Dahm et al., 2022; Jackson et al., 2003; Kraeutner et al., 2016a,2016b; Land et al., 2016; Wohldmann et al., 2007, 2008), and sometimes even more effective than physical practice (e.g., Wohldmann et al., 2008). While imagery practice of coordination tasks can affect kinematics as well (Gatti et al., 2013; Gentili et al., 2010; Kraeutner et al., 2020b), it seems to particularly improve perceptual-cognitive aspects of action control (e.g., memory: Frank et al., 2014; planning: Frank et al., 2016; for a review, see Moran & O’Shea, 2020). Finally, strength tasks can profit from imagery practice suggesting that improvements particularly relate to central improvements and cognitive elements of strength tasks (Lebon et al., 2010; Reiser et al., 2011; Yue & Cole, 1992).

From a perceptual-cognitive scaffolding perspective, imagery practice is superior for tasks with a high cognitive component, because scaffolding through repeated action effect anticipation directly improves task performance, since successful task performance primarily depends on cognitive aspects, while the motor aspects of the task are trivial (or learnt). Instead, for complex tasks with high motor components that require coordination between body parts or new coordination patterns (e.g., toe abduction; Mulder et al., 2004), the perceptual-cognitive scaffolding does not necessarily lead to changes in overt behavior, and leads to motor learning only if a link between anticipated effects and the related coordination pattern exists (see as well next point).

**(2) On the influence of the imager’s skill level**: ***Imagery is more effective in terms of motor performance for imagers that have some experience with the task, but functional changes on perceptual-cognitive levels of action organization can be found in novices.***

Although evidence exists that imagery practice can be effective both in novices and skilled athletes (Blair, 1989 in Hall et al., 1992; Suinn, 1980; Toth et al., 2020; Wrisberg & Ragsdale, 1979), meta-analyses indicate that the impact of imagery on performance and learning is more effective when the imager has some experience with the task (Driskell et al., 1994; Toth et al., 2020). The majority of studies comparing imagery practice to physical practice in novices report small to no effects for imagery practice relative to those resulting from practice via overt action (Frank et al., 2014; Ingram et al., 2019; Kraeutner et al., 2020b; Ruffino et al., 2021; for a review, see Simonsmeier et al., 2020). This shows that, when imagery practice is performed in isolation of overt action, the magnitude of overt learning and/or performance improvements is minimal. Finally, it has been shown that in case of true novices, imagery practice does not lead to any motor performance gains. To rule out any transfer effects from similar tasks, Mulder and colleagues (2004) used a toe abduction task which was completely new for participants. Their findings nicely illustrate that imagery practice has no effect on motor performance when the imager has absolutely no prior experience with the task.

From a neuroimaging perspective, prior work has shown that expertise modulates imagery-based brain activation patterns (Chang et al., 2010; Kraeutner et al., 2018; Milton et al., 2007); more diffuse patterns of activity were observed when skilled athletes imagined a sports-specific skill incongruent with their expertise (e.g., a volleyball player imagining a basketball free throw) than when they imagined a sports-specific skill congruent with their expertise (e.g., a volleyball player imagining an overhand serve). Here, these more focal patterns were attributed to experts being able to access a well-established representation of the skill during imagery (relating back to experts’ elaborate representation structures, see Schack & Mechsner, 2006), requiring less cognitive resources to generate/access the representation and thus greater perceptual fluency of the action (Kraeutner et al., 2018; Milton et al., 2007).

While imagery does not lead to any or only little improvement of motor performance of a task (Mulder et al., 2004; Toth et al., 2020), studies looking at underlying perceptual-cognitive changes show that novices develop functional representational networks of perceptual-cognitive units during imagery practice that do not necessarily transfer into better motor performance, and thus do not (yet) become visible in terms of improvements in overt action (Frank et al., 2014). For instance, Frank and colleagues (2014) have shown that three sessions of imagery practice led to more elaborate representation structures of the golf putt in novices’ memory, whilst this cognitive advantage did not transfer into improved motor performance during this early stage of learning and without any task execution. Interestingly, other work using serial reaction time(-like) tasks (i.e., tasks with high cognitive components) in novice performers have shown practice via imagery to result in improvements in performance like that observed for practice via overt action (Dahm et al., 2022; Kraeutner et al., 2016a, 2016b). Considering this finding alongside those of Frank and colleagues (2014), who showed imagery practice was inferior to physical practice for tasks with high motor components, suggests imagery practice may be particularly effective in facilitating perceptual changes (as opposed to movement per se) in tasks with high motor components, especially when the imager has no prior experience.

The superiority of higher level of experience with the task can be explained, as the perceptual-cognitive scaffold used during imagery is already linked to the action itself, transferring cognitive into motor improvements. Instead, novices start building a perceptual-cognitive scaffold as a result of imagery practice, while this is not yet linked to their motor repertoire and thus requires task execution to be adjusted and to come into effect.

**(3) On transfer effects.** ***Transfer after imagery practice is possible and sometimes greater compared to physical practice; changing perceptual components in transfer tasks particularly impairs learning by way of imagery practice﻿.***

Work employing transfer tasks further suggests that imagined action induces a perceptual-cognitive scaffold. Per the motor simulation theory, wherein imagined and overt action are functionally equivalent, performance on transfer tasks should also be similar. Yet, this is not always the case. Prior research on transfer effects has suggested that imagery practice engenders a representation of movement that is independent of effector (i.e., effector independent learning) relative to physical practice (Healy et al., 2012; Wohldmann et al., 2008). For instance, Wohldmann and colleagues (2008, Exp. 2) found that transfer to the unpracticed hand was more pronounced in imagery practice than in physical practice, highlighting what they called the “mental practice superiority effect”. Considering that representations are more effector-independent after imagery practice than after physical practice, it might be that they are even more flexible after imagery practice than after physical practice. Such tasks have been used to show that imagery practice leads to greater inter-manual transfer than physical practice (Land et al., 2016; Wohldmann et al., 2008). However, imagery practice does not always result in superior transfer, and sometimes the effects are similar to physical practice (Dahm et al., 2022), which may depend on the specific task and/or effectors involved.

In works such as those described above, the task goal and perceptual components remained the same, although the effector used to perform the task is changed. In contrast, performance on perceptual transfer tasks, i.e., tasks in which the effector used to perform the task, and thus the motor command, remain the same but the perceptual components and requirements of the task have changed, is disrupted following imagery practice vs. physical practice (Ingram et al., 2016). For instance, Ingram and colleagues (2016) manipulated either the effector or the perceptual cue, showing that a change in perceptual cue impacted performance following imagery practice greater than a change in effector, with the opposite finding observed for performance following training via overt action. This further supports that imagery practice helps establish a perceptual-cognitive scaffold of the task to be learnt, and if the perceptual components of the scaffold do not correspond to the perceptual components of the task to be performed (i.e., in the case of a perceptual transfer task), then performance is disrupted.

Taken together, this evidence supports the notion of a perceptual-cognitive scaffold: transfer is possible particularly when tasks share action effects, and such effects are more readily transferred from one effector to another.

**(4) On the influence imagery instruction.** ***Imagery practice is more effective when imagined from a task-relevant perspective and when focusing on the task-relevant modalities.***

Looking at instructions how to perform imagery during imagery practice, both the perspective from which we imagine and the (quasi)sensory modality we focus on during imagery of a motor action, influence the impact imagery practice can have on performance and learning (Hall et al., 1992; Mahoney & Avener, 1977; White & Hardy, 1995; for a review, see Morris et al., 2005).

Findings from neuroscience indicate that an internal perspective (‘looking through one’s own eyes’) recruits more motor-related areas compared to an external perspective (‘looking from a camera’s perspective’) (Hétu et al., 2013; Mizuguchi et al., 2016; Stinear et al., 2018). Along these lines and according to applied models, it has been suggested that one should imagine from one’s own perspective (Holmes & Collins, 2001). Evidence from studies comparing perspective across different tasks indicates that the preference and impact of the perspective depends highly on the sport and the task (e.g., Spittle & Morris, 2012; White & Hardy, 1995).

While imagery is multimodal (Lacey & Lawson, 2013; for details on multimodal action imagery, see Krüger et al. 2022), visual and kinesthetic aspects of the imagined action are of particular importance when it comes to movement (Cumming & Williams, 2012). Kinesthetic imagery produces more muscular activity (Harris & Robinson, 1986) and recruits more motor-related areas compared to visual imagery (Guillot et al., 2009; Stinear et al., 2005), which may be indicative that imagining the kinesthetic aspects of a motor action is superior to any other modality of imagery when it comes to performing or learning a motor action. Evidence exists, however, that the impact of modality during imagery practice depends on the modalities of the task to be learnt (Féry, 2003; Toussaint et al., 2010). For instance, in a visual-spatial drawing task performance was better after visual imagery practice than after kinesthetic imagery practice (Féry, 2003, Exp. 1), while in a bimanual coordination task participants’ performance was better after kinesthetic imagery practice than after visual imagery practice (Féry, 2003, Exp. 2).

The superiority of perspective and modalities critical for the task to be learnt can be explained from an effect-based perspective, as imagery leads to perceptual-cognitive scaffolding of multimodal action effects, with the scaffold being naturally constructed from the task-relevant perceptual components. Imagery practice therefore is most effective when it includes the task-relevant modalities imagined from an appropriate perspective.

**(5) On the influence of combinations of imagined and overt action for practice and practice schedules.** ***While imagery practice does not adhere to scheduling principles known from physical practice, it can add to learning if combined with physical practice.***

Motor learning research shows that practicing in a variable manner (e.g., golf putts of different length) leads to better learning than practicing in a specific manner (e.g., golf putts of same length). Similarly, random practice (e.g., practicing different putt lengths randomly mixed) is more beneficial than blocked practice (e.g., practicing different putt lengths by practicing the short one first for a block of trials, then the long one etc.; Schmidt et al., 2019). Such scheduling effects after physical practice, could not been found after imagery practice, neither for task variability (Coelho et al., 2012) nor for random practice (Overdorf et al., 2004), pointing again to differences in the two types of learning.

Furthermore, combinations of imagery practice and physical practice can lead to similar or even greater improvements in motor performance compared to physical practice alone, even when the number of practice trials is held constant (McBride & Rothstein, 1979; Simonsmeier et al., 2020). From testing different rates of imagery practice relative to physical practice in a grasping task, imagery practice has proven as beneficial as overt practice when learners imagined as many as (50%) or even more (75%) trials relative to overt trials during practice, particularly in more complex tasks (Allami et al., 2008). Preliminary work seeking to leverage the more perceptual nature of imagined action reported front-loading 5 days of blocked imagery practice prior to 5 days of physical practice resulted in greater performance improvements compared to the reverse order (Kraeutner et al., 2020a). This ordering effect of imagery practice preceding physical practice suggests that forming a perceptual-cognitive scaffold via imagery prior to overt action may facilitate learning.

Finally, dynamic forms of imagery (dynamic motor imagery/ dMI; for a review, see Guillot et al., 2021) in comparison to static forms of imagery without any overt action has proven beneficial for performance and learning: It may be that moving during imagery helps activate stored action effects, and as such might add to representational refinement, possibly by linking the (minimal) action to its (imagined) effects. In sum, both superadditive effects from combinations of imagery and physical practice and differences in scheduling effects between imagery practice and physical practice cannot be explained by theories that consider imagined action and overt action equivalent. This again points to a different role for imagery practice in the learning of motor actions.

From an effect-based point of view on the combined practice, the perceptual-cognitive scaffold built through action effect anticipation during imagery practice is fed with actual feedback from overt action during physical practice. In this way, achieved effects perceived through feedback after physical practice can be linked to the goal-oriented perceptual-cognitive scaffold (Frank, 2014; Frank et al., 2014). Instead, Practice trials without any actual feedback help novices to focus on goal-oriented action effect anticipation, and thus to build a functional perceptual-cognitive scaffold during imagery practice (Frank, 2014). Accordingly, scheduling effects from physical practice research cannot be found after imagery practice, as these require and are explained by making use of constant actual feedback.

## Understanding learning via imagery – Quo vadis?

In this position paper, we advocate a perceptual-cognitive approach to imagery practice effects and suggest perceptual-cognitive scaffolding as a potential mechanism that drives learning by way of imagery.

According to the perceptual-cognitive scaffolding idea proposed, action effects are being imagined by anticipating sensory consequences of the action during imagery. If performed repeatedly during imagery practice, this imagery of action effects leads to scaffolding of anticipated action effects, being stored as part of one’s action representation in long-term memory to guide future action. This ‘perceptual’ learning (i.e., perceptual-cognitive scaffolding) transfers into ‘motor’ learning (i.e., changes in overt behavior) – or not – depending on various factors such as type of task, skill level, imagery perspective and modality, or transfer (for details, see rationales on influential factors (1)–(5)).

While the perceptual-cognitive scaffolding hypothesis is grounded in evidence covering a wide range of phenomena observed in imagery practice, future research is required to further develop and test this idea. Specifically, open questions to be addressed range from the role of the following:(1) feedback to drive perceptual-cognitive scaffolding (e.g., prediction of action consequences; Rieger et al. in this issue); (2) executive functions during perceptual-cognitive scaffolding (Glover & Baran, 2017), (3) simulation in perceptual-cognitive scaffolding (Jeannerod, 2001); (4) inverse and/ or forward models during imagery and imagery practice (Bach et al., 2022; Rieger et al. in this issue); to (5) the manipulation of aspects of the movement representation such as its (multi)sensory quality of imagery (for a review, see Krüger et al., 2022), and (6) the exploration of superadditive effects of action observation as a truly sensory format to the imagery process (see Eaves et al., 2022).

## Footnotes

1 Please note that in order to prevent potential confusion of terms, we refer to imagined vs. overt action in this manuscript, with both action states involving (quasi)planning and (quasi)execution stages. In other words, we consider execution stages as part of both imagined actions [imagined execution, no overt movement] and overt actions [real execution, overt movement]. Likewise, we refer to imagery practice (interchangeably used with imagery training, mental practice) and physical practice.

2 Quasi-perceptual effects during imagery denote effects that are being reconstructed from memorial information (i.e., imagined effects), in contrast to effects that are being perceived during overt action (i.e., perceived effects).

3 In contrast to Heuer’s view (1985, 1989) that imagery practice from an ideomotor view can be explained by the minimal peripheral effects of imagery in the muscles (and thus is not a valid explanation), we highlight an ideomotor account to be currently the most appropriate explanation to explain imagery practice effects through its focus on action effects, and the guiding role of perceptual-cognitive aspects during goal-directed action.
